# Early Predictors of Steroid Failure in Acute Severe Ulcerative Colitis: A Retrospective Study With the Evaluation of the Admission Model for Intensification of Therapy in Acute Severe Colitis (ADMIT-ASC) Score

**DOI:** 10.7759/cureus.109276

**Published:** 2026-05-20

**Authors:** Othmane Merzouki, Saad Mliyahe, Houda Meyiz, Imane El Fadli, Manal Aziz, Yasmine Ghannami, Hassan Ouaya, Aicha Akjay, Ihssan Mellouki

**Affiliations:** 1 Gastroenterology and Hepatology Department, Mohammed VI University Hospital, Tangier, MAR

**Keywords:** acute severe ulcerative colitis, anti-tumor necrosis factor, gastroenterology and endoscopy, predictive scoring, steroid refractory

## Abstract

Background and aims: Acute severe ulcerative colitis (ASUC) is a life-threatening gastroenterological emergency in which early identification of patients at risk of corticosteroid failure is critical for timely therapeutic escalation. This study aimed to identify early predictive factors of corticosteroid failure and to evaluate the performance of the Admission Model for Intensification of Therapy in Acute Severe Colitis (ADMIT-ASC) score in predicting therapeutic failure in a North African cohort.

Methods: This was a retrospective, single-center study. Patients hospitalized for ASUC between September 2021 and January 2026 with complete data for ADMIT-ASC calculation were included (n = 87). The primary outcome was corticosteroid failure, defined as the requirement for rescue anti-TNF therapy or emergency colectomy during the same index hospitalization. Disease severity was assessed using the Truelove and Witts (TW) criteria, the Lichtiger score, the Ulcerative Colitis Endoscopic Index of Severity (UCEIS), the Oxford criteria (Travis score), and the ADMIT-ASC score. Fisher's exact test was used when expected cell counts were below 5; the chi-square (χ²) test otherwise.

Results: Of 87 patients included, 53 (60.9%) achieved clinical remission under intravenous corticosteroids; 27 patients (31.0%) required rescue therapy during the index hospitalization: 21 received anti-TNF rescue therapy (primarily infliximab 5 mg/kg), and 6 (6.9%) underwent emergency surgical colectomy; and the remaining 7 (8.1%) patients had incomplete outcome documentation in the hospital records, were retained in the denominator, and were excluded from the primary outcome analysis. The presence of more than two additional TW criteria (including fever, tachycardia, anemia, and elevated CRP beyond stool frequency) was the strongest admission predictor (χ² = 11.44, OR = 7.27 (95% CI: 2.37-22.31), p = 0.001). The ADMIT-ASC score ≥ 3 was significantly associated with failure (χ² = 4.34, OR = 3.92 (95% CI: 1.20-12.80), p = 0.037; sensitivity of 33.3% (95% CI: 18.6-52.2%), specificity of 88.7% (95% CI: 77.4-94.7%)). In a Firth penalized logistic regression, including both admission predictors, both remained independently associated with failure: adjusted OR 7.37 (95% CI: 2.35-23.12) for additional TW criteria and 5.10 (95% CI: 1.44-18.07) for ADMIT-ASC ≥ 3 (p = 0.012). The Lichtiger score ≥ 11 showed a trend toward significance without reaching it (p = 0.114). The UCEIS ≥ 7 was not significantly associated with failure (p = 0.631). The Travis score at day three remained the strongest predictor overall (OR = 153.1; p < 0.001).

Conclusions: Traditional clinical markers at admission - particularly the number of additional TW criteria - remain highly effective predictors of corticosteroid failure. The ADMIT-ASC score is a promising adjunct for structured early risk stratification, with a high specificity (88.7%), complementing rather than replacing traditional clinical assessment and the Travis score at day three.

## Introduction

Acute severe ulcerative colitis (ASUC) is a life-threatening complication of inflammatory bowel disease (IBD), classically defined by the clinico-biological criteria of Truelove and Witts (TW) [1] and requiring urgent hospitalization. Intravenous corticosteroids remain the cornerstone of first-line treatment [2], but 30-40% of patients fail to respond and require rescue therapy or emergency colectomy [3].

Identifying non-responders early matters beyond avoiding unnecessary steroid exposure. In the setting of severe colonic inflammation, the intestinal mucosal barrier is severely disrupted, leading to substantial fecal loss of protein-bound drugs; the concurrent hypoalbuminemia compounds this by reducing infliximab's protein-bound fraction, together driving accelerated drug clearance precisely in those patients who will ultimately need it most [4]. This means that identifying likely non-responders early is not just about avoiding a few days of ineffective corticosteroids - it is about maximizing the pharmacokinetic window of rescue biologic therapy. There is also a surgical dimension: in patients destined for colectomy, delayed escalation is associated with greater operative morbidity. Several clinical, biological, and endoscopic parameters have been described to identify these patients [5], including the Oxford criteria (Travis score) [6] and the Ho index [7], both validated day three tools whose late timing limits proactive decision-making. Recent systematic reviews have catalogued the full landscape of predictive scores developed to address this gap [8].

The Admission Model for Intensification of Therapy in Acute Severe Colitis (ADMIT-ASC) score was developed and validated in an international cohort as a promising admission-based composite tool integrating CRP, albumin, and Ulcerative Colitis Endoscopic Index of Severity (UCEIS). Because disease phenotypes and biological responses may vary geographically, external validation in a North African cohort - where the epidemiology of IBD may differ from Western Europe - is of scientific and clinical relevance. This study aimed to identify early predictive factors of steroid failure and to evaluate the performance of the ADMIT-ASC score in our Moroccan cohort.

## Materials and methods

Study design and setting

We conducted a retrospective, observational, single-center study at the Department of Gastroenterology and Hepatology of Mohamed VI University Hospital, Tangier, Morocco. Patients hospitalized for ASUC between September 2021 and January 2026 were identified from hospital medical records. The study was conducted in accordance with the Declaration of Helsinki. Due to its retrospective nature and exclusive use of anonymized routine clinical data, formal ethical committee approval was not required. As a single-center retrospective study, results should be interpreted with caution regarding generalizability to other healthcare settings.

Study population and inclusion criteria

All patients aged 15 years or older hospitalized for ASUC were initially screened. The diagnosis of ASUC was established by the modified TW criteria defined by the presence of at least six bloody stools per day associated with at least one sign of systemic toxicity: temperature of > 37.8°C, heart rate of > 90 beats per minute, hemoglobin of < 10.5 g/dL, erythrocyte sedimentation rate (ESR) of >30 mm/hour, or CRP of > 30 mg/L [1]. For this analysis, only patients with complete data for ADMIT-ASC calculation (CRP, albumin, and UCEIS all available) were included (n = 87). All inaugural flare cases were confirmed as ulcerative colitis by histological examination of endoscopic biopsies; patients with Crohn's colitis or indeterminate colitis were excluded. Patients with confirmed infectious colitis (*Cytomegalovirus* (CMV) or *Clostridioides difficile*) or contraindications to corticosteroid therapy were also excluded.

Data collection

Demographic, clinical, biological, and endoscopic data were systematically extracted from hospital medical records. Biological parameters included serum CRP, albumin, hemoglobin, and ESR. Flexible sigmoidoscopy or colonoscopy was performed within 24-48 hours of admission in all patients.

Scoring systems

The following validated open-access scoring tools were recorded:

Truelove and Witts (TW) criteria: Stool frequency and systemic toxicity signs. The number of additional systemic criteria (beyond stool frequency) was also recorded [1].

Lichtiger score (LS): It is a validated clinical activity index for severe UC comprising eight items: stool frequency, nocturnal diarrhea, visible blood in stools, fecal incontinence, abdominal pain or cramping, general well-being, abdominal tenderness, and need for antidiarrheal drugs. A score ≥10 defines severe disease [9].

UCEIS: It is an endoscopic severity score comprising three domains: vascular pattern, bleeding, and erosions/ulcers. Scores range from 0 to 8, with scores of 7-8 indicating severe disease. The UCEIS is freely available in the published literature [10].

Oxford criteria (Travis score): Day three predictor: positive if > 8 stools/day or 3-8 stools with CRP > 45 mg/L [6].

ADMIT-ASC score: It integrates three parameters measurable at admission: CRP level, serum albumin, and UCEIS score, generating a composite score (range 0-3) to stratify risk of corticosteroid failure. A score ≥ 3 identifies patients at high risk for treatment escalation [11] (Table 1).

**Table 1:** ADMIT-ASC score components and scoring criteria ADMIT-ASC = Admission Model for Intensification of Therapy in Acute Severe Colitis; UCEIS = Ulcerative Colitis Endoscopic Index of Severity Each parameter is scored 0 (absent/below threshold) or 1 (present/above threshold). A total score of 3 indicates the highest level of risk for corticosteroid failure, as validated by Adams et al. [11].

Parameter	Threshold	Points awarded	Data source
C-reactive protein (CRP)	≥ 100 mg/L at admission	1	Blood test at admission
Serum albumin	≤ 25 g/L at admission	1	Blood test at admission
UCEIS	≥ 7 on admission endoscopy	1	Flexible sigmoidoscopy within 24-48h
Total score range	0-3	-	-
High-risk threshold	Score ≥ 3	-	-

Primary outcome

Corticosteroid failure was defined as the requirement for rescue anti-TNF therapy (primarily infliximab) or emergency surgical colectomy during the same index hospitalization. Two patients who experienced ASUC recurrence within one year of discharge are described separately in the results and were not included in the primary outcome analysis.

Statistical analysis

Statistical analysis was performed using Statistical Product and Service Solutions (SPSS, version 28.0; IBM SPSS Statistics for Windows, Armonk, NY). Continuous variables are expressed as median (IQR). Comparisons used the Mann-Whitney U test for continuous variables. For categorical variables, Fisher's exact test was applied when any expected cell count was < 5 (i.e., sex); the chi-square (χ²) test was used for all other categorical variables. Test statistics (U or χ²), OR with 95% CI, and p-values are reported throughout. Sensitivity, specificity, positive predictive value (PPV), and negative predictive value (NPV) are reported with 95% CIs calculated using the Wilson method. A two-tailed p < 0.05 was considered statistically significant. Given the limited number of outcome events (n = 27), a Firth penalized logistic regression was performed as a two-variable exploratory multivariate model including the two significant admission predictors identified in univariate analysis. Firth's method was chosen because it provides reliable estimates in settings of small sample size and potential quasi-complete separation, conditions that would bias standard maximum likelihood estimation.

## Results

Baseline characteristics

Of the 87 patients included, the median age was 32 years (range: 15-85), with a slight male predominance (47 patients, 54.0%). Median disease duration was three years (IQR: 1-6). Sixty patients (69.0%) had pancolitis (E3), 15 (17.2%) left-sided colitis (E2), and 11 (12.6%) proctitis or distal colitis (E1). Thirty-seven patients (42.5%) were experiencing an inaugural flare, all confirmed as ulcerative colitis on histological examination. Regarding treatment at admission: 47 patients (54.0%) were on 5-ASA, 9 (10.3%) on azathioprine, 7 (8.0%) on biologic or combination therapy, and 24 (27.6%) had no background treatment. Table 1 presents the full baseline characteristics and univariate analysis.

**Table 2:** Baseline characteristics and univariate analysis of the predictors of corticosteroid failure (n = 87) * = p < 0.05, ¹ = admission-based predictors that were significant in univariate analysis, and ² = strongest overall predictive performance (positive Travis score at day 3); † = analyzed on patients with complete TW data (n = 86); § = Fisher's exact test (minimum expected cell count < 5); χ² = used for all other categorical variables; TW = Truelove and Witts; CRP = C-reactive protein; IQR = interquartile range; UCEIS = Ulcerative Colitis Endoscopic Index of Severity; ADMIT-ASC = Admission Model for Intensification of Therapy in Acute Severe Colitis; OR = odds ratio; CI = confidence interval

Variable	Responders (n = 53)	Non-Resp. (n = 27)	Test statistic	OR (95% CI)	p-value
Age, median years (range)	33 (15-85)	34 (16–77)	U = 727	-	0.911
Male sex, n (%)§	31 (58.5%)	11 (40.7%)	Fisher’s exact	0.46 (0.17-1.24)	0.16
Disease duration, median years (IQR)	3 (1-6)	4 (1-6)	U = 901	-	0.701
Pancolitis (E3), n (%)	35 (66.0%)	20 (74.1%)	χ² = 0.23	1.47 (0.52-4.16)	0.632
Inaugural flare, n (%)	20 (37.7%)	14 (51.9%)	χ² = 0.94	1.78 (0.70-4.52)	0.333
Lichtiger score ≥ 11, n (%)	26 (49.1%)	19 (70.4%)	χ² = 2.49	2.47 (0.88-6.90)	0.114
>2 additional TW criteria, n (%)†	6 (11.3%)	13 (48.1%)	χ² = 11.44	7.27 (2.37-22.31)	0.001^*1^
CRP at admission, median mg/L (IQR)	37.5 (13.8-126.2)	89.5 (17.5-174.2)	U = 316	-	0.203
Albumin at admission, median g/L (IQR)	36.8 (33.0-40.0)	40.0 (35.0-41.0)	U = 241	-	0.165
Stool frequency/day, median (IQR)	10 (6-12)	10 (10-14)	U = 277	-	0.069
UCEIS ≥ 7 (severe), n (%)	23 (43.4%)	14 (51.9%)	χ² = 0.23	1.40 (0.55-3.56)	0.631
ADMIT-ASC score ≥ 3, n (%)	6 (11.3%)	9 (33.3%)	χ² = 4.34	3.92 (1.20-12.80)	0.037^*1^
Positive Travis score (day 3), n (%)	4/53 (7.5%)	25/27 (92.6%)	χ² = 52.4	153.1 (25.8-909)	< 0.001^*2^
Stool frequency > 8/day at day 3, n (%)	2/38 (5.3%)	10/19 (52.6%)	χ² = 14.4	20.0 (3.8-105)	< 0.001^*1^
CRP at day 3, median mg/L (IQR)	12 (5-23)	44 (16-98)	U = 216	-	0.003^*1^

Corticosteroid response and rescue therapy

Of the 87 patients, 53 (60.9%) achieved clinical remission under intravenous corticosteroids; 27 patients (31.0%) required rescue therapy during the index hospitalization: 21 received anti-TNF rescue therapy (primarily infliximab 5 mg/kg), and six (6.9%) underwent emergency surgical colectomy; and the remaining 7 (8.1%) patients had incomplete outcome documentation in the hospital records, were retained in the denominator, and were excluded from the primary outcome analysis. All six colectomies were emergency procedures performed during the index hospitalization; none were elective. The corticosteroid failure rate of 31.0% is consistent with contemporary literature [3,5,12]. The emergency colectomy rate of 6.9% compares favorably with historical series [2], reflecting proactive biologic rescue therapy use.

Performance of the ADMIT-ASC score

The ADMIT-ASC score distribution in the 87 patients was as follows: score 0 in one patient (1.1%), score 1 in 32 (36.8%), score 2 in 39 (44.8%), and score 3 in 15 (17.2%). Among the 15 patients with a score ≥ 3, 9 (60.0%) failed corticosteroids compared to 18/72 (25.0%) with a score < 3 (χ² = 4.34, OR = 3.92 (95% CI: 1.20-12.80), p = 0.037). The diagnostic performance of the ADMIT-ASC score ≥ 3 was as follows: sensitivity of 33.3% (95% CI: 18.6-52.2%), specificity of 88.7% (95% CI: 77.4-94.7%), PPV of 60.0% (95% CI: 35.7-80.2%), and NPV of 72.3% (95% CI: 60.4-81.7%). All 95% CIs were calculated using the Wilson method.

The Travis score at day three showed superior overall performance: sensitivity of 92.6% (95% CI: 76.6-97.9%), specificity of 92.5% (95% CI: 82.1-97.0%), PPV of 86.2% (95% CI: 69.4-94.5%), and NPV of 96.1% (95% CI: 86.8-98.9%).

Multivariate analysis

Given the small number of outcome events (n = 27), a full multivariate logistic regression would have been underpowered and at risk of overfitting. We therefore performed a Firth penalized logistic regression, which is specifically designed to provide reliable estimates in the setting of small samples and potential quasi-complete separation. The two significant admission predictors identified in univariate analysis - presence of more than two additional TW criteria and ADMIT-ASC score ≥ 3 - were entered simultaneously as covariates. This analysis was conducted as a two-variable exploratory model and should be interpreted accordingly.

Both variables remained independently associated with corticosteroid failure after mutual adjustment (Table 2). The ADMIT-ASC score ≥ 3 retained independent predictive value with an adjusted OR of 5.10 (95% CI: 1.44-18.07, p = 0.012), and the presence of more than two additional TW criteria showed an adjusted OR of 7.37 (95% CI: 2.35-23.12, p = 0.001). The overall model fit was significant (likelihood ratio test p = 0.00004).

**Table 3:** Firth penalized logistic regression: independent predictors of corticosteroid failure (n = 87, events = 27) *p < 0.05. †Firth penalized logistic regression, both variables entered simultaneously (n = 87, 27 events). Likelihood ratio test p = 0.00004. TW = Truelove and Witts; ADMIT-ASC = Admission Model for Intensification of Therapy in Acute Severe Colitis; OR = odds ratio; CI = confidence interval; χ² = chi-square statistic from univariate analysis

Variable	Univariate OR (95% CI)	Adjusted OR (95% CI)†	χ²	p-value
>2 additional TW criteria	7.27 (2.37-22.31)	7.37 (2.35-23.12)	11.44	0.001*
ADMIT-ASC score ≥3	3.92 (1.20-12.80)	5.10 (1.44-18.07)	4.34	0.012*

## Discussion

This retrospective single-center study evaluated early predictors of corticosteroid failure in 87 patients hospitalized for ASUC with complete data for ADMIT-ASC calculation. The corticosteroid failure rate of 31.0% is consistent with contemporary literature: the systematic review by Turner et al. [12] confirmed a stable failure rate of approximately 34% across three decades, and more recent analyses have shown no meaningful decline despite therapeutic advances [3,5]. The emergency colectomy rate of 6.9% compares favorably with historical series [2], reflecting the proactive use of anti-TNF rescue therapy.

Among admission-based predictors, the number of additional TW criteria - encompassing systemic markers, such as fever, tachycardia, anemia, elevated ESR, and elevated CRP beyond the stool frequency threshold [1] - was the strongest predictor of failure (OR = 7.27, p = 0.001), consistent with population-based data from Wanderås et al. [13]. The more systemic toxicity criteria a patient accumulates at admission, the less likely the colonic inflammation is to respond to corticosteroids alone - a clinically intuitive finding that is now quantified in our cohort. The Lichtiger score [9] showed a trend toward significance without reaching it (p = 0.114): a type II error due to limited sample size cannot be excluded, given the observed difference in proportions (70.4% vs 49.1%). The UCEIS [10] was not significantly associated with failure (p = 0.631). This is best understood as a consequence of population selection rather than a failure of the score itself: all patients in this cohort met the TW criteria for severe colitis, concentrating the endoscopic severity distribution in the upper range of the scale where fine gradations carry less discriminative weight. In more heterogeneous populations, including moderate disease, the UCEIS would be expected to perform differently. Traditional clinical markers therefore remain highly effective and immediately actionable at admission.

The ADMIT-ASC score ≥ 3 [11] was significantly associated with failure in univariate analysis (p = 0.037, OR = 3.92), and retained independent predictive value after adjustment for additional TW criteria in the Firth penalized regression (adjusted OR = 5.10 (95% CI: 1.44-18.07), p = 0.012). The fact that the adjusted OR for ADMIT-ASC was higher than the unadjusted OR suggests that the two predictors capture genuinely different dimensions of disease severity rather than overlapping information - additional TW criteria reflect systemic toxicity detectable at the bedside, while ADMIT-ASC integrates biological inflammatory burden and endoscopic severity into a structured composite. Together, they describe a more complete picture of the patient at risk than either does alone. As noted by Angkeow et al. and Kayal et al., admission scores and day three scores address different decision windows and are most effective when used sequentially [5,8]: the ADMIT-ASC identifies high-risk patients at entry for early preparation of escalation, while the Travis score [6] provides the most accurate prediction at day three (OR = 153.1, NPV = 96.1%), confirming or ruling out the need for rescue therapy with high confidence.

CRP and albumin at admission did not reach statistical significance as individual predictors in our cohort. This likely reflects the fact that, in a population of uniformly severe patients, the between-patient variability in these markers is insufficient to discriminate outcomes when they are examined in isolation. Gibson et al. [14] reported that a CRP/albumin ratio > 0.85 predicted non-response, with a relative risk of 1.63 - a composite approach that, by combining both parameters, improves the signal-to-noise ratio. Similarly, the meta-analysis by Zheng et al. confirmed elevated CRP and hypoalbuminemia as significant colectomy predictors when analyzed in combination [15]. Recent reviews of ASUC management [16] and single-center series [17] have further highlighted that biological predictors perform best when integrated into composite scoring rather than assessed independently, reinforcing the conceptual basis of the ADMIT-ASC.

The pharmacokinetic rationale for early risk stratification is important: severe colonic inflammation produces significant fecal protein loss that accelerates infliximab clearance. Goyal et al. described how proactive admission-based stratification is reshaping ASUC management [18]. Evidence from Antunes et al. [19] supports intensified infliximab induction in high-risk patients, a concept formally validated by the PREDICT-UC trial [20], which showed that accelerated induction was superior to standard dosing for corticosteroid-refractory ASUC. This pharmacokinetic vulnerability is well documented: mucosal barrier disruption leads to fecal drug loss, and the concurrent hypoalbuminemia reduces protein-bound drug concentrations, both accelerating infliximab clearance in the acute setting [4]. Identifying high-risk patients via ADMIT-ASC at admission could enable earlier preparation of such regimens without waiting for the day three assessment.

The study's main limitations are its retrospective single-center design and modest sample size, which generated wide confidence intervals for some estimates and precluded a full multivariate model beyond the two-variable Firth regression. External validation in prospective North African multicenter cohorts is warranted to confirm these findings.

## Conclusions

In this cohort of 87 patients with ASUC and complete ADMIT-ASC data, traditional clinical markers - particularly the presence of more than two additional TW criteria at admission (adjusted OR = 7.37) - were the most powerful admission predictors of corticosteroid failure and remain a highly effective and immediately actionable bedside tool. The ADMIT-ASC score is a promising adjunct for structured early risk stratification, with a clinically useful specificity of 88.7% and an independent association with failure confirmed by Firth penalized logistic regression (adjusted OR = 5.10, p = 0.012). It should be seen as complementary to the established Travis score at day three rather than as a replacement. Prospective multicenter studies in North African populations are needed to definitively validate its integration into standardized management algorithms.
